# Decreased soluble Nogo-B in serum as a promising biomarker for Parkinson's disease

**DOI:** 10.3389/fnins.2022.894454

**Published:** 2022-07-26

**Authors:** Hongming Liang, Wenyuan Guo, Honghu He, Hui Zhang, Qiongyu Ye, Qingxin Zhang, Jiajia Liao, Yuefei Shen, Jin Wang, Yousheng Xiao, Chao Qin

**Affiliations:** ^1^Department of Neurology, The First Affiliated Hospital of Guangxi Medical University, Nanning, China; ^2^Department of Neurology, The First People's Hospital of Yulin, The Sixth Affiliated Hospital of Guangxi Medical University, Yulin, China; ^3^Department of Neurology, The First Affiliated Hospital of Guangzhou Medical University, Guangzhou, China; ^4^Department of Rehabilitation Medicine, The First Affiliated Hospital of Guangxi Medical University, Nanning, China; ^5^Department of Rehabilitation Medicine, The First People's Hospital of Yulin, The Sixth Affiliated Hospital of Guangxi Medical University, Yulin, China

**Keywords:** *NUS1*, biomarkers, α-Synuclein, Parkinson's disease, Nogo-B receptor

## Abstract

**Background:**

Recently, the neurite outgrowth inhibitor-B (Nogo-B) receptor has been reported as a novel candidate gene for Parkinson's disease (PD). Nogo-B receptors need to combine with soluble Nogo-B to exert their physiological function. However, little is known about the relationship between serum soluble Nogo-B and PD.

**Methods:**

Serum levels of sNogo-B and α-Synuclein (α-Syn) were measured in a cohort of 53 patients with PD and 49 healthy controls with the ELISA kit method.

**Results:**

Serum sNogo-B level is significantly lower in the PD group than that in healthy controls and is negatively correlated with UPDRS-III score (*p* = 0.049), H&Y stage (*p* = 0.0108) as well as serum α-Syn level (*p* = 0.0001). The area under the curve (AUC) of serum sNogo-B in differentiating patients with PD from controls was 0.801 while the AUC of serum α-Syn was 0.93. Combining serum sNogo-B and α-Syn in differentiating patients with PD from HC presented higher discriminatory potential (AUC = 0.9534).

**Conclusion:**

Decreased serum sNogo-B may be a potential biomarker for PD. Lower Nogo-B level reflects worse motor function and disease progression of PD. Serum sNogo-B is of added value to serum α-Syn panel in distinguishing PD from controls. Future studies are needed to confirm in larger samples and different populations.

## Introduction

Parkinson's disease (PD) is one of the most common neurodegenerative disorders. It has affected over 1% of people beyond 65 years old around the world (Tanner, 1996; Langston, 1998). PD is pathologically characterized by abnormal accumulation of α-Synuclein (α-Syn) in dopaminergic neurons from the substantia nigra of the midbrain (Wong and Krainc, 2017). The diagnosis of PD mainly relies on clinical characteristics at present, but pathological examination results in a post-mortem study demonstrated only 76% accuracy in the clinical diagnosis of PD (Hughes et al., 1992), which raises concern that clinical diagnosis of PD may lead to many misdiagnoses and delays the early identification of PD.

Nowadays, there are many studies on diagnostic biomarkers of PD such as blood (Zhao et al., 2018), cerebrospinal fluid (Parnetti et al., 2019), and imaging (Pyatigorskaya et al., 2014; Satue et al., 2016). A typical biomarker is α-Syn, but the results are inconsistent due to the presence of α-Syn in various body fluids and tissue together with the presence of many α-Syn variants (Atik et al., 2016). Therefore, there is still a lack of effective biomarkers for early diagnosis of PD and there is an urgent need to identify novel, sensitive, and reliable biomarkers in order to discover new diagnostic methods for patients with PD.

Recently reported as a novel candidate gene for PD, the *NUS1* codes the Neurite outgrowth inhibitor-B (Nogo-B) receptor (Guo et al., 2018; Chen et al., 2020). Functional studies in Drosophila have shown that loss of *NUS1* reduces the fly's motor function, dopamine levels as well as the number of dopaminergic neurons, and induces apoptosis in the fly's brain (Guo et al., 2018). In addition, studies have shown that Nogo-B is involved in many key cellular processes such as cholesterol transportation (Harrison et al., 2009), dolichol synthesis and protein N-glycosylation (Harrison et al., 2011), vascular remodeling and genesis, tumorgenesis (Wang et al., 2013; Pula et al., 2014; Zhao et al., 2015) and especially neural development (Guo et al., 2018; Zhang et al., 2020). Nogo-B is supposed to inhibit the growth and reconnection of synapses and affect the expression of proinflammatory cytokine derived from microglia in central nervous system diseases (Fang et al., 2015). Furthermore, the amino terminus of Nogo-B is a soluble peptide (soluble Nogo-B or sNogo-B) which can be secreted into body fluids like serum and CSF (Rodriguez-Feo et al., 2007; Hernandez-Diaz et al., 2019) and is capable of binding to the Nogo-B receptor (Zhang et al., 2020). However, little is known about the relationship between serum soluble Nogo-B and PD. In this study, we aimed to investigate the role of serum sNogo-B for PD and its association with PD's severity, especially motor symptoms. Moreover, serum α-Syn levels were measured to explore its association with sNogo-B. We also tested if serum sNogo-B can differentiate PD from healthy controls as a single marker or combining with serum α-Syn.

## Materials and methods

### Participants

We recruited 53 patients with PD (including early and advanced patients) and 49 age-and sex-matched healthy controls from the Department of Neurology of First Affiliated Hospital of Guangxi Medical University between August 2017 and September 2019. PD's diagnostic criteria followed the United Kingdom PD Society Brain Bank (Hughes et al., 1992). Exclusion criteria were: other neurological diseases except for PD, accompanied by tumor or acute infectious diseases, severe cognitive impairment (MMSE score < 24), or other conditions that may interfere with the clinical evaluation. Every patient was diagnosed by two professional neurologists. The demographic and clinical characteristics of all subjects are shown in Table 1. The study was approved by the ethics committee of The First Affiliated Hospital of Guangxi Medical University. Written informed consents were obtained from all of the participants.

**Table 1:** Demographic and clinical features of patients with PD and healthy control.

	**PD group (*****n*** **=** **53)**	**HC group (*****n*** **=** **49)**	***p*** **-value**
Age (years)	58.14 ± 10.82	56.88 ± 10.17	0.255
Age of onset (years)	54.68 ± 12.65	–	–
Disease duration (years)	3.17 ± 2.93	–	–
sNogo-B (pg/ml), mean ± SD	107.12 ± 27.62	291.57 ± 27.44	<0.0001
α-Syn (pg/ml), mean ± SD	4,664.36 ± 1,049.91	2,354.27 ± 1,177.95	<0.0001
UPDRS III score, mean ± SD	34.63 ± 18.77	–	–
Hoehn and Yahr stage, mean ± SD	2.12 ± 0.89	–	–
MMSE score, mean ± SD	28.84 ± 1.61	28.83 ± 0.99	0.163
NMSS score, mean ± SD	25.00 ± 15.683	–	–
Levodopa equivalent dose (mg/d), mean ± SD	324.78 ± 37.92	–	–

*PD, Parkinson's Disease; HC, healthy controls; SD, Standard deviation; UPDRS III, Unified Parkinson's Disease Rating Scale III; MMSE, Mini-Mental State Examination; NMSS, Non-Motor Symptoms Scale*.

### Clinical evaluation

All patients recruited were evaluated on the day of the sample collection with the Unified Parkinson's Disease Rating Scale III (MDS-UPDRS III) (Goetz et al., 2007) and the Hoehn and Yahr staging scale (H&Y). MMSE score and the Non-Motor Symptoms Scale (NMSS) (Chaudhuri et al., 2007) were used to evaluate the cognitive function and the non-motor symptom of patients with PD. Levodopa equivalent dose (LED) was calculated as well (Tomlinson et al., 2010).

### Sampling and biological assays

All recruited participants' venous blood samples were collected with vacuum tubes on the morning of the patients' visit after an overnight fast. Blood samples were centrifuged at 3,000 g for 15 min at 4°C and then aliquoted and stored at −80°C until analysis. LEGEND MAX™ Human Nogo-B ELISA Kit (Detectable levels of 95% sample ranging from 30 to 468 pg/ml) were used to determine sNogo-B levels in the serum of patients with PD. In order to verify our determination with prior studies, the levels of α-Synuclein in serum were determined by Abcam Human α-Synuclein ELISA Kit (Detectable range: 281 to 3,200 pg/ml). The samples were diluted if necessary during the assay. All of the above procedure was carried out in strict accordance with the instructions.

### Statistical analysis

All statistical analyses were performed with IBM SPSS Statistics 23 and all data were tested for normality of distribution (*p* > 0.05). Comparisons between groups were made using the Chi-squared test for categorical data, and independent samples *t*-test or the Mann–Whitney *U*-test for continuous variables. A receiver operating characteristic curve was drawn to calculate the area under the curve for sensitivity and specificity. Pearson's or Spearman's rank correlation analyses were conducted to evaluate the correlation between levels of sNogo-B, α-Synuclein, and clinical variables.

## Results

**Figure 1:**
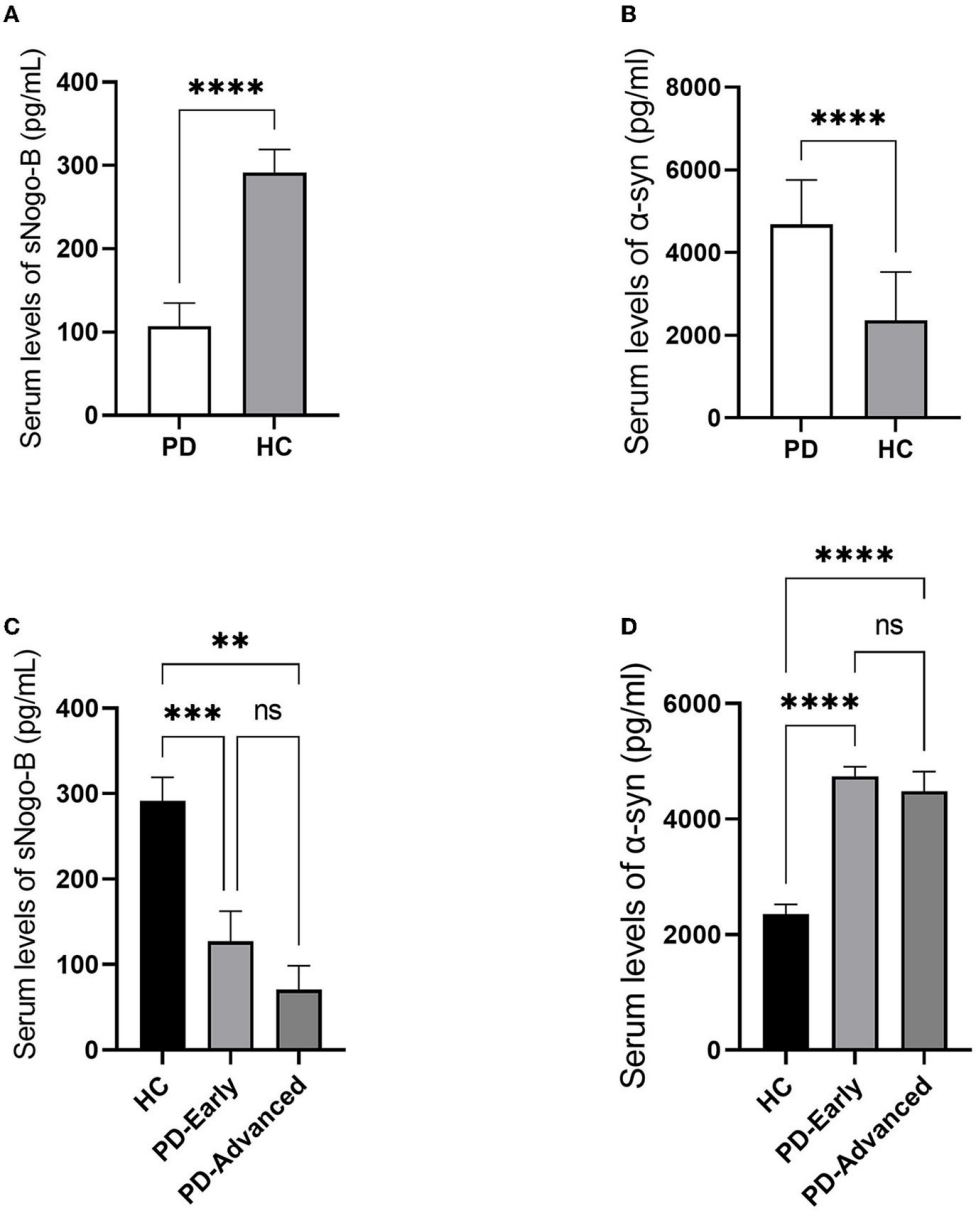
Comparisons of Serum levels of sNogo-B and α-Syn in patients with PD and HC. **(A)** Serum levels of sNogo-B in patients with PD and HC. Patients with PD presented lower serum levels of sNogo-B than healthy control individuals (*p* < 0.0001); **(B)** Serum levels of α-Syn in patients with PD and HC. Patients with PD presented higher serum levels of α-Syn than healthy control individuals (*p* < 0.0001); **(C)** Serum levels of sNogo-B in early and advanced patients with PD and HC. No significant difference was observed between serum sNogo-B levels of early and advanced patients with PD (*p* = 0.521); **(D)** Serum levels of α-Syn in early and advanced patients with PD and HC. No significant difference was observed between serum α-Syn levels of early and advanced patients with PD (*p* = 0.857); PD, Parkinson's disease; α-Syn, α-Synuclein; HC, healthy controls. **, *p* < 0.01; ***, *p* < 0.001; ****, *p* < 0.0001; ns, not significant.

Patients with PD and healthy controls were matched in age and gender. Serum sNogo-B levels were observed lower in PD group than that in HC group (107.12 ± 27.62 pg/ml vs. 291.57 ± 27.44 pg/ml, *p* < 0.0001) while serum α-Syn levels were observed higher in PD group than that in HC group (4,664.36 ± 1,049.91 pg/ml vs. 2,354.27 ± 1,177.95 pg/ml, *p* < 0.0001), as illustrated in Figure 1. Subgroup analysis according to separate stage showed that there was no significant difference between early and advanced groups in serum sNogo-B levels (127.04 ± 35.48 pg/ml vs. 70.43 ± 28.18 pg/ml, *p* = 0.521) as well as α-Syn levels (4,723.71 ± 157.29 pg/ml vs. 4,484.6 ± 290.25 pg/ml, *p* = 0.857), which was shown in Figure 1.

**Figure 2:**
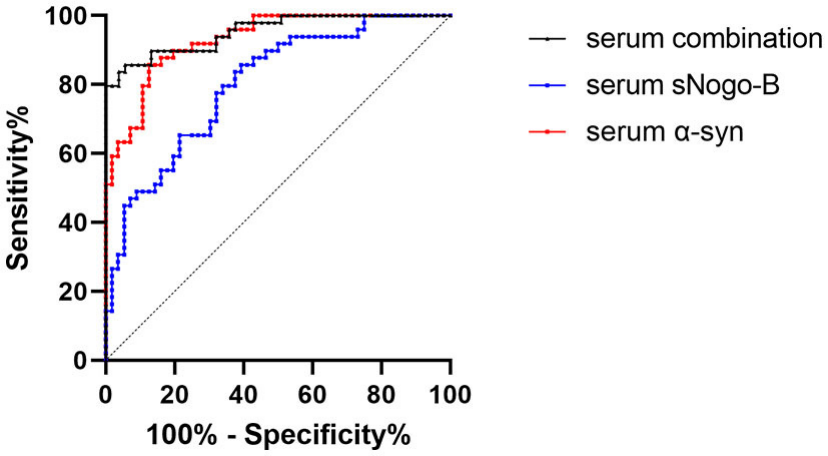
ROC curve of serum levels of sNogo-B (AUC = 0.801, *p* < 0.0001), serum α-Syn (AUC = 0.93, *p* < 0.0001) and combination of them (AUC = 0.9534, *p* < 0.0001). Both sNogo-B and α-Syn in serum presented significant diagnostic value of PD; Combination of sNogo-B and α-Syn presented the higher diagnostic value of PD.

According to the receiver operating characteristic curve analysis, the area under the curve (AUC) of serum sNogo-B in differentiating patients with PD from HC was 0.801 and the cutoff value was 209.09 pg/ml; Sensitivity and specificity were 91.1 and 49%, respectively. While AUC of serum α-Syn in differentiating patients with PD from HC was 0.93 and the cutoff value was 3,712.67 pg/ml. Sensitivity and specificity were 86.8 and 85.7%, respectively. The AUC of combining serum sNogo-B and α-Syn in differentiating patients with PD from HC was 0.9534 and sensitivity and specificity were 94.3 and 85.7%, respectively (Figure 2).

**Figure 3:**
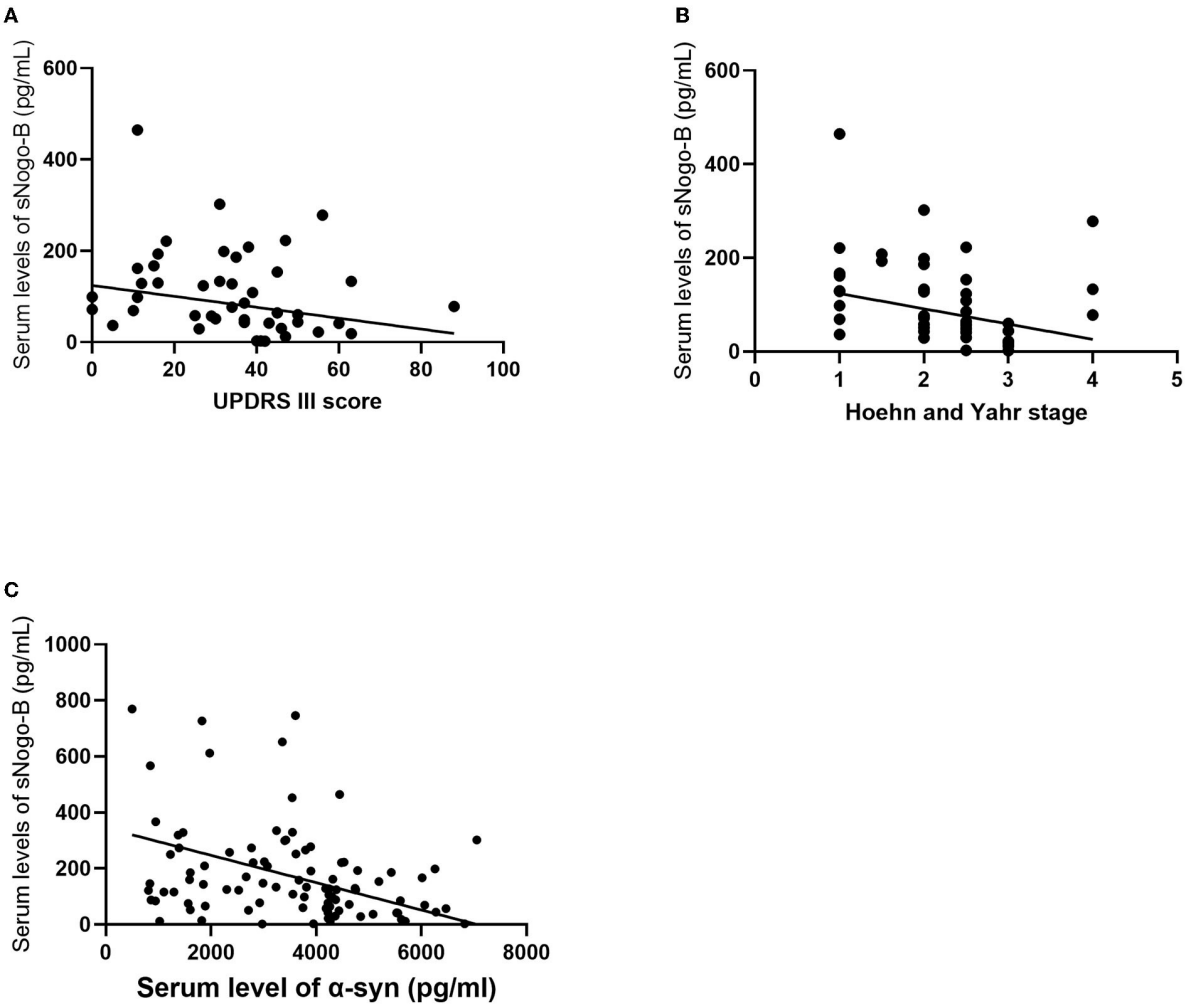
Correlations analysis of serum sNogo-B and clinical characteristics. **(A)** Serum sNogo-B level negatively correlated with UPDRS III score (*r* = −0.264, *p* = 0.049); **(B)** Serum sNogo-B level negatively correlated with Hoehn and Yahr stage (*r* = −0.344, *p* = 0.0108); **(C)** Serum sNogo-B level negatively correlated with serum α-Syn level (*r* = −0.365, *p* = 0.0001).

Pearson or Spearman correlation analysis showed that serum sNogo-B level was mildly negatively correlated with UPDRS III scores (*r* = −0.264, *p* = 0.049) and negatively correlated with the Hoehn and Yahr stage (*r* = −0.344, *p* = 0.0108) and serum α-Syn level (*r* = −0.365, *p* = 0.0001), respectively (Figure 3). Nevertheless, we failed to observe any significant correlations between levels of serum sNogo-B and NMSS scores (25 ± 15.683, *r* = −0.134, *p* = 0.338) as well as levodopa equivalent dose (324.78 ± 37.92 mg/d, *r* = −0.092, *p* = 0.497).

## Discussion

Soluble neurite outgrowth inhibitor-B is a circulating isoform of full-length Nogo-B. Previous studies reveal that Nogo-B plays an important role in many diseases like atherosclerosis (Rodriguez-Feo et al., 2007), cancer, childhood epilepsy, and neurodegenerative disease (Eckharter et al., 2015; Zhang et al., 2020), and its potential as a novel candidate gene of Parkinson's disease (Guo et al., 2018). However, there is little evidence for altered sNogo-B in PD to date. To the best of our knowledge, we are the first to find out that serum sNogo-B levels in patients with PD decreased significantly compared with healthy controls. Considering the role of Nogo-B/NgBR in promoting axonal branching (Eckharter et al., 2015), sNogo-B may act as protecting antibody and potentially take effect in neural repairment and even treatment of PD. Moreover, it was found that NgBR is highly expressed in the cell body and axon of sensory neurons, and the interaction between Nogo-B expressed by Schwann cells and NgBR could further promote axonal branching (Eckharter et al., 2015) while PI3K/ Akt signal pathway is involved in promoting neuron survival, axon growth and axon branching (Huang et al., 2017; Wang et al., 2019; Zhu et al., 2019). It is suggested that sNogo-B may take part in the pathogenesis of PD by down-regulating the expression of related proteins in the PI3K/Akt signaling pathway by inhibiting the Nogo-B/NgBR signal axis.

Serum α-Syn levels in PD presented higher than that in HCs, which is consistent with the results of some previous research (Park et al., 2011; Atik et al., 2016). In addition, serum α-Syn levels in PD were negatively correlated with serum sNogo-B levels, which indicates the connection of sNogo-B and α-Syn. It's reported that aggregation of misfolded and degenerative α-Syn caused by endoplasmic reticulum dysfunction is involved in the pathophysiological process of PD (Costa et al., 2020). In physiologic conditions, the endoplasmic reticulum (ER) is involved in the quality control of intracellular proteins. ER-associated degradation (ERAD) selectively delivers degenerative or misfolded proteins to the proteasome for degradation, thus preventing abnormal proteins from cellular secretion (Ruggiano et al., 2014; Schwarz and Blower, 2016). In the case of ER-proteasome dysfunction, α-Syn aggregation may affect the endoplasmic reticulum and result in ER stress (Bernal-Conde et al., 2019; Costa et al., 2020). In addition, it has been found that ER to Golgi trafficking and mitochondrial damage in PD models can be reversed by compounds reducing α-Syn toxicity (Su et al., 2010). Moreover, Nguyen et al. have also reviewed that ER-mitochondria-lysosome dysfunction is involved in the pathophysiological process of PD (Nguyen et al., 2019). Since Nogo-B is a member of the endoplasmic reticulum protein family (Zhang et al., 2020), it is suggested that down-regulation of Nogo-B may lead to ER - proteasome and ER - lysosome dysfunction, which leads to the decline of α-syn clearance ability, thus participating in the pathogenesis of PD. This hypothesis needs to be explored by further experimental studies.

Furthermore, the association between clinical features and serum sNogo-B levels was explored. Lower serum sNogo-B was found to be associated with severer motor symptoms and higher Hoehn and Yahr stage. Although serum sNogo-B levels could not distinguish between early and advanced patients by now, the trend of decreasing levels of sNogo-B in advanced PD is foreseeable, which deserves future investigation in larger samples. As loss of Nogo-B reduces the fly's movement ability, dopamine levels, and the number of dopaminergic neurons (Guo et al., 2018), sNogo-B might potentially reflect the severity of motor symptoms and disease progression in patients with PD. Since levodopa equivalent dose has no effect on serum sNogo-B level, it indicates the potential for therapeutic targeting of the dysregulated Nogo-B in PD.

Interestingly, the area under the curve of serum sNogo-B in differentiating patients with PD from HC is lower than the AUC of α-Syn. We combined serum sNogo-B and α-Syn in order to improve the diagnostic value for PD and the results showed that the AUC of the combination was higher as expected. It's worth noting that α-Syn may not act as a diagnostic indicator for PD as α-Syn levels in serum or plasma varies in different studies because of the existence of variant of α-Syn as well as the influence of erythrocyte in α-Syn (Atik et al., 2016; Barkovits et al., 2020).

Of course, there are limitations to this study. First, the sample size is small and patients with PD were mainly in the early stage, thus further studies of larger sample numbers are needed. Second, since both α-Syn and Nogo-B function in the brain, the decreased sNogo-B level in the periphery did not directly reflect the levels in the brain. Therefore, the measurement of sNogo-B and α-Syn in cerebrospinal fluid level might be important to establish the connection between Nogo-B and PD. Third, in this present study, we focused on the difference between PD and healthy people, related movement disorders like essential tremor, multiple system atrophy, or progressive supranuclear palsy were not involved, which might be distinct from PD and should be explored in the future.

## Conclusion

Our study suggests that decreased serum sNogo-B may be a potential biomarker for PD. The lower Nogo-B level reflects the worse motor function of PD. Serum sNogo-B is of added value to serum α-Syn panel in distinguishing PD from controls. Future studies are needed to confirm in larger samples and different populations.

## Data availability statement

The raw data supporting the conclusions of this article will be made available by the authors, without undue reservation.

## Ethics statement

The studies involving human participants were reviewed and approved by the Ethics Committee of The First Affiliated Hospital of Guangxi Medical University. The patients/participants provided their written informed consent to participate in this study.

## Author contributions

HL and WG carried out the experiments and wrote the manuscript. HH carried out the experiments as well. HZ, QY, QZ, JL, YS, and JW collected and collated the data. CQ and YX designed the study, offered support of funding, and revised the paper. All authors agreed to be accountable for the content of the work and contributed to the article and approved the submitted version.

## Funding

This study was supported by the Innovation Project of Guangxi Graduate Education (No. YCSW2019112), Guangxi Natural Science Foundation Program (No. 2019GXNSFDA185008), and Guangxi Medical and Health Appropriate Technology Development and Application Project (No. S2019095).

## Conflict of interest

The authors declare that the research was conducted in the absence of any commercial or financial relationships that could be construed as a potential conflict of interest. The reviewer PX declared a shared affiliation with the author WG to the handling editor at the time of review.

## Publisher's note

All claims expressed in this article are solely those of the authors and do not necessarily represent those of their affiliated organizations, or those of the publisher, the editors and the reviewers. Any product that may be evaluated in this article, or claim that may be made by its manufacturer, is not guaranteed or endorsed by the publisher.
